# 

**Published:** 1883-08

**Authors:** 


					﻿Whole Number,
CCLV.
AUGUST, 1883.
Number i,
Volume XXIII.
THE
BUFFALO
MediGal and Surgical Journal.
EDITORS:
THOS. I.OTHROP, M.
A. R. DAVIDSON, M. D.,
R. W. VAN PEYMA, M. ».
Published by the Medical Journal Association.
No. B WEST CHIPPEWA ST.
Entered at the Post-Office at Buffalo, N. Y., as Second-class Mail Matter.
				

